# National study of United States emergency department visits for acute pancreatitis, 1993–2003

**DOI:** 10.1186/1471-227X-7-1

**Published:** 2007-01-22

**Authors:** Peter J Fagenholz, Carlos Fernández-del Castillo, N Stuart Harris, Andrea J Pelletier, Carlos A Camargo

**Affiliations:** 1 Department of Surgery, Massachusetts General Hospital, Harvard Medical School, Boston, MA, USA; 2 Department of Emergency Medicine, Massachusetts General Hospital, Harvard Medical School, Boston, MA, USA

## Abstract

**Background:**

The epidemiology of acute pancreatitis in the United States is largely unknown, particularly episodes that lead to an emergency department (ED) visit. We sought to address this gap and describe ED practice patterns.

**Methods:**

Data were collected from the National Hospital Ambulatory Medical Care Survey between 1993 and 2003. We examined demographic factors and ED management including medication administration, diagnostic imaging, and disposition.

**Results:**

ED visits for acute pancreatitis increased over the study period from the 1994 low of 128,000 visits to a 2003 peak of 318,000 visits (p = 0.01). The corresponding ED visit rate per 10,000 U.S. population also increased from 4.9 visits (95%CI, 3.1–6.7) to 10.9 (95%CI, 7.6–14.3) (p = 0.01). The average age for patients making ED visits for acute pancreatitis during the study period was 49.7 years, 54% were male, and 27% were black. The ED visit rate was higher among blacks (14.7; 95%CI, 11.9–17.5) than whites (5.8; 95%CI, 5.0–6.6). At 42% of ED visits, patients did not receive analgesics. At 10% of ED visits patients underwent CT or MRI imaging, and at 13% of visits they underwent ultrasound testing. Two-thirds of ED visits resulted in hospitalization. Risk factors for hospitalization were older age (multivariate odds ratio for each increasing decade 1.5; 95%CI, 1.3–1.8) and white race (multivariate odds ratio 2.3; 95%CI, 1.2–4.6).

**Conclusion:**

ED visits for acute pancreatitis are rising in the U.S., and ED visit rates are higher among blacks than whites. At many visits analgesics are not administered, and diagnostic imaging is rare. There was greater likelihood of admission among whites than blacks. The observed race disparities in ED visit and admission rates merit further study.

## Background

Acute pancreatitis is a relatively common condition, but the incidence throughout the Western world varies widely. For example, hospital admission rates of 1.5 cases per 10,000 in Southern England and 7.3 per 10,000 in Finland have been reported in various European studies. In addition, investigators have described varied demographic characteristics and treatment patterns for different European populations [1-4]. No European studies have specifically examined emergency department (ED) visit rates for acute pancreatitis. In the U.S., no national studies have described the epidemiology of acute pancreatitis from any perspective (i.e. hospital admission rates, ED visit rates, or visits to all healthcare facilities), or the initial evaluation and treatment of pancreatitis patients in U.S. EDs. This national study of U.S. ED visits over an 11-year period thus stands to provide the most complete epidemiologic picture to date of pancreatitis in the U.S., offering insight into recent trends in ED visits, the overall burden of the disease in the U.S., and the demographic characteristics of patients.

## Methods

Data from the ED component of the National Hospital Ambulatory Medical Care Survey (NHAMCS) from 1993–2003 were combined for analysis [5-15]. The NHAMCS is a 4-stage probability sample of visits to non-institutional general and short stay hospitals, excluding Federal, military, and Veterans Administration hospitals, located in the 50 States and the District of Columbia [16,17]. The NHAMCS is conducted annually and covers geographic primary sampling units, hospitals within primary sampling units, EDs within hospitals, and patients within EDs. Trained hospital staff collect data during a randomly assigned 4-week data period for each sampled hospital, approximately once every 15 months [18]. Review of data collection is performed by a U.S. Bureau of Census field supervisor. Quality control includes computer checks to assess inconsistencies with value ranges, a two-way 10-percent independent procedure for medical and drug coding, and adjudication by the National Center for Health Statistics (NCHS) for ambiguous or illegible responses for fields including reasons for visit and diagnosis. The non-response rate for most items is less than 5%, and error rates are less than 2% for items that require medical coding [17]. When the data collection forms are completed, they are sent to Constella Group Inc., Durham, North Carolina where they are coded by experienced personnel using the *International Classification of Disease, Ninth Revision, Clinical Modification *(ICD-9-CM). National estimates are obtained through use of assigned patient visit weights and are rounded to the nearest thousand. A multistage estimation procedure consists of inflation by reciprocals of the sampling selection probabilities, adjustment for non-response, and a population weighting ratio adjustment. The NHAMCS data form is devoid of patient identifying characteristics. A more detailed description of the NHAMCS data collection and estimation procedures is available for review at the National Hospital Ambulatory Medical Care Survey (NHAMCS) Public-use Data Files web page and in the technical notes section of each year's NHAMCS Emergency Department Summary [19,20].

The NHAMCS allows for specification of up to three physician diagnoses. In this study, we first identified all visit records with the physician diagnosis ICD-9-CM 577.0 (acute pancreatitis) in any position and determined estimated ED visits and visit rates. For all subsequent analysis we then excluded visit records if ICD-9-CM 577.0 appeared only as the third-listed diagnosis or if ICD-9-CM 577.1 (chronic pancreatitis) appeared as a physician diagnosis in any diagnostic position. This was done to minimize possible problems from miscoding of chronic pancreatitis exacerbations as episodes of acute pancreatitis, and to eliminate equivocal diagnoses of acute pancreatitis.

We examined ED visits by patient age, sex, race, Hispanic ethnicity, and insurance status and by hospital metropolitan statistical area (MSA) status and region (Northeast, Midwest, South and West). MSA and U.S. region categories represent standardized geographical divisions defined by the U.S. Bureau of the Census; essentially, a hospital in a MSA is urban [21,22]. Hispanic ethnicity was not imputed by the NHAMCS from 1997–2002, and thus data on Hispanic ethnicity are not available for those years. U.S. visit rates were computed using mid-year age, sex, race, ethnicity, and metropolitan status specific population estimates from the U.S. Census Bureau; all rates were reported per 10,000 individuals per year for the U.S. resident population. Investigators also re-examined primary results for visit rates using the civilian population, as recommended by NCHS, and results were similar (data not shown). Overall average annual rates for the entire study period, where reported (such as in Table 1), were calculated by dividing the total number of estimated ED visits by the sum of the midyear estimates for each of the eleven years of the study. ED management focused on medications, diagnostic imaging, and ED disposition (e.g., hospital admission). Disease severity data are limited but include urgency at triage. To keep analyses between earlier and later years consistent, we coded visits that occurred after a change in coding in 1997 (1997–2003), as "urgent/emergent" if immediacy to be seen was recorded as "less than 15 minutes" or "15–60 minutes," and as "non-urgent" if recorded as ">1–2 hours" or longer. From 1993–94 up to five medications were recorded per visit, from 1995–2002 up to six medications were recorded per encounter, and in 2003 up to eight medications were recorded per encounter, with medications coded as per published NCHS definitions [23-25]. All recorded medications were considered for analysis. Therapeutic class of medication (eg. "antibiotic" or "analgesic") was based on the National Drug Code Directory.

**Table 1:** Emergency Department Visits for Acute Pancreatitis in the U.S. by Patient and Hospital Characteristics; 1993–2003.

	**n**	**Cumulative Number of Visits**	**95% Confidence Interval**		**Average Annual Visit Rate per 10,000 US Population**	**95% Confidence Interval**		**Average Annual Visit Rate per 10,000 ED Visits**	**95% Confidence Interval**	
Overall (acute pancreatitis in any diagnostic position, without exclusions)	649	2,235,000	1,983,000	2,487,000	7.4	7.0	7.6	20.2	18.0	22.0

Overall (after imposing exclusion criteria)	595	2,052,000	1,821,000	2,282,000	6.8	6.1	7.6	18.5	16.4	20.6
										
Age Group (years)										
<10	8	nc*	nc	nc	nc	nc	nc	nc	nc	nc
10–19	10	nc	nc	nc	nc	nc	nc	nc	nc	nc
20–29	69	234,000	167,000	300,000	5.2	3.7	6.6	12.6	9.0	16.2
30–39	104	288,000	220,000	355,000	5.5	4.2	6.9	16.7	12.8	10.7
40–49	153	508,000	402,000	615,000	10.6	8.4	12.8	37.4	29.5	45.2
50–59	92	346,000	251,000	441,000	10.3	7.5	13.1	39.4	28.6	50.2
60–69	76	269,000	189,000	349,000	11.0	7.7	14.3	40.1	28.2	52.1
70–79	48	212,000	135,000	289,000	11.2	7.1	15.3	30.7	19.5	41.8
80+	35	136,000	80,000	192,000	13.0	7.7	18.4	22.0	12.9	31.1
										
Sex										
Female	265	954,000	797,000	1,110,000	6.2	5.2	7.2	16.3	13.6	18.9
Male	330	1,098,000	945,000	1,251,000	7.5	5.4	7.6	21.0	15.3	21.3
										
Race										
White	407	1,424,000	1,225,000	1,624,000	5.8	5.0	6.6	16.8	14.5	19.2
Black	164	559,000	453,000	666,000	14.7	11.9	17.5	24.0	19.4	28.6
Other	24	nc	nc	nc	nc	nc	nc	nc	nc	nc
										
Ethnicity^†^										
Hispanic	64	198,000	130,000	266,000	5.7	3.7	7.6	20.8	13.7	28.0
Non-Hispanic	419	1,484,000	1,286,000	1,683,000	5.6	4.8	6.3	21.2	18.4	24.0
										
U.S. Region										
Northeast	145	414,000	306,000	521,000	7.2	5.3	9.0	19.0	14.1	23.9
Midwest	122	441,000	343,000	539,000	6.3	4.9	7.8	15.6	12.2	19.1
South	187	740,000	613,000	866,000	7.0	5.8	8.2	18.1	15.0	21.2
West	141	458,000	331,000	584,000	6.8	5.0	8.7	23.0	16.6	29.3
										
Metropolitan Status										
Metropolitan	526	1,692,000	1,466,000	1,917,000	7.0	6.1	8.0	19.5	16.9	22.1
Non-metropolitan	69	360,000	228,000	492,000	5.8	3.7	8.0	15.0	9.5	20.5

*Not calculable because sample <30 or relative standard error >30%

^†^21% sample missing

We performed data management and analysis using STATA 9.0 (StataCorp, College Station, TX). A masked ultimate cluster sample design was used to estimate variance. NCHS considers an estimate to be unreliable if it has a relative standard error (SE) of more than 30%. In addition, estimates based on fewer than 30 records are considered inherently unreliable, regardless of their SE. For the current analysis, we determined point estimates and 95% confidence intervals (CIs) for ED visits – both absolute numbers and population rates – as well as for visits by patient, age, sex, race, ethnicity, geographic region, and MSA status. Visits also were analyzed for frequency of hospital admission, analgesic administration (including narcotics), computed tomography (CT) or magnetic resonance imaging (MRI) use, and ultrasound use. CT, MRI, and ultrasound use were not recorded in 1993–94, so these results describe practice patterns from 1995–2003. Pearson's chi-square was used to assess differences between groups. Chi-square for trend was used to evaluate trends over the 11-year period. Visits were combined into 2-year groups (with the exception of 2003) to determine whether trends existed for total visits, or by sex or race. Multivariate logistic regression was used to evaluate independent predictors of hospital admission. Two-sided p-values of less than 0.05 were considered statistically significant. Our study was conducted with the approval of the Massachusetts General Hospital Institutional Review Board.

## Results

The 1993–2003 NHAMCS ED dataset included 304,697 ED visits, of which 649 were coded as acute pancreatitis. These visits represent an estimated 2,235,000 (95%CI, 1,983,000 – 2,487,000) ED visits for "acute pancreatitis" and an overall average annual ED visit rate of 7.4 (95%CI, 6.6–8.3) per 10,000 U.S. population for the entire 11-year study period. Exclusion of sample records that were also coded as "chronic pancreatitis" and sample records with acute pancreatitis as the third-listed diagnosis left 595 visits, which are the focus of all subsequent analyses. These visits represent an estimated 2,052,000 (95%CI, 1,822,000 – 2,282,000) ED visits for acute pancreatitis and an overall rate of 6.8 (95%CI, 6.1–7.6) ED visits per 10,000 U.S. population for the entire 11-year study period. At triage, 76% (95%CI, 71–81%) were considered urgent/emergent.

Demographic characteristics of ED visits for acute pancreatitis are shown in Table 1. In brief, population rates were positively associated with age, with a significant increase at the fifth decade (age 40–49) that was relatively stable thereafter. The ED visit rate per 10,000 U.S. population among blacks (14.7; 95%CI, 11.9–17.5) was more than double that among whites (5.8; 95%CI, 5.0–6.6). Compared to all other ED visits acute pancreatitis visits were more likely to be made by males (p = 0.01). Rates did not differ by gender, urban setting, or U.S. region. Insurance status for ED visits with acute pancreatitis was similar to that of all other ED visits.

**Figure 1:**
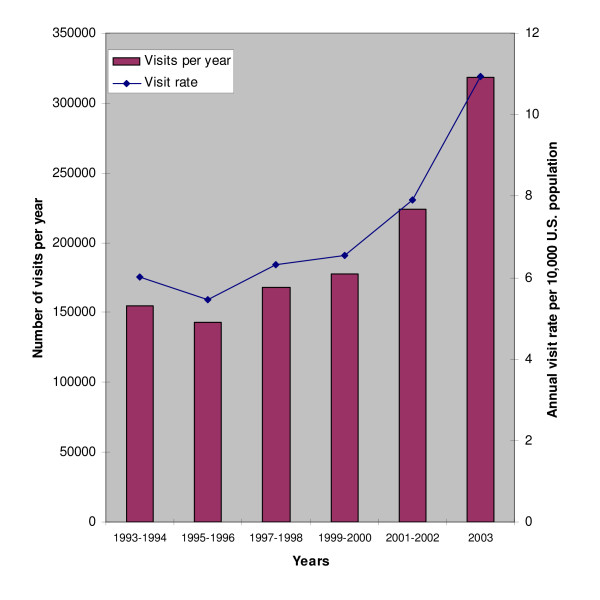
**Total Emergency Department Visits and Visit Rates for Acute Pancreatitis in the U.S. 1993–2003**. Annual ED Visits and Visit Rates for acute pancreatitis in the U.S. 1993–2003. Data are grouped in two-year blocks with the exception of 2003.

**Figure 2:**
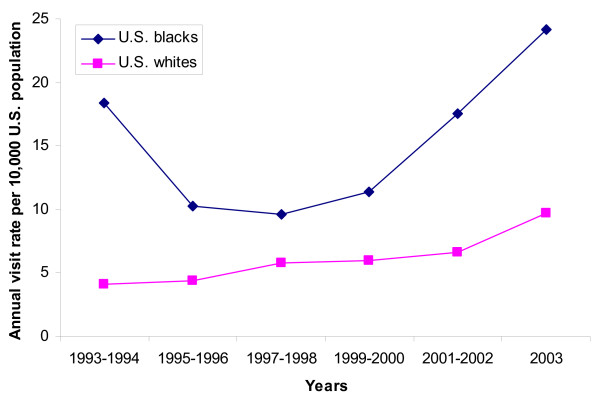
**Emergency Department Visit Rates for Acute Pancreatitis by Race, 1993–2003**. Visit rates for blacks and whites with acute pancreatitis in U.S. Emergency Departments from 1993–2003. Figures are 2-year annual averages, with the exception of 2003. P for trend = 0.03 for whites, indicating a significant upward trend during the study period. P for trend = 0.24 for blacks, indicating that there was no significant upward trend.

Looking at time trends over the 11-year study period, there was a significant upward trend in both the absolute numbers and the population rates for ED visits for acute pancreatitis (Figure 1; both p for trend < 0.05). The nadir year for total visits was 1994 with 128,000 visits (95%CI, 81,000 – 175,000) and the peak was 2003 with 318,000 visits (95%CI, 221,000 – 415,000). The population rate was also lowest in 1994 at 4.9 visits per 10,000 individuals (95%CI, 3.1–6.7) and peaked in 2003 at 10.9 (95%CI, 7.6–14.3). The observed increases were fairly uniform across various basic demographic groups, including adults age 18 and older (p = 0.04), males (p = 0.09), females (p = 0.09), and whites (p = 0.03). Although rates for blacks were consistently higher than those for whites across all years (Figure 2), we did not observe a statistically significant increase for blacks over the 11-year time period (p = 0.24).

Analgesics were administered during 58% (95%CI, 53–63%) of ED visits for acute pancreatitis. Overall, narcotic analgesics were administered during 40% (95%CI, 35–46%) of all ED visits, so when analgesics were given they were most often narcotics. Blacks and whites were equally likely to receive analgesics (51.1% vs. 60.6%; p = 0.10). Antibiotics were administered during 9% (95%CI, 6–11%) of all visits. In terms of imaging, CT scanning or MRI were performed during 10% (95%CI, 7–13%) of visits, while ultrasound was performed 13% (95%CI, 9–17%) of the time. These numbers were too small to allow analysis of trends in imaging. Overall, 65% (95%CI, 60–69%) of ED visits for acute pancreatitis resulted in admission to the hospital.

Table 2 provides results of the multivariate logistic regression model of hospital admission. The only independent predictors of admission were older age (multivariate odds ratio of 1.5 for each increasing decade) and white race (multivariate odds ratio of 2.3). The immediacy with which the patient should be seen, as perceived at triage, had a non-significant positive association with admission. By contrast, gender, ethnicity, insurance type, urban location, and U.S. region were not associated with hospital admission.

**Table 2:** Multivariate Model of Hospital Admission After an Emergency Department Visit for Acute Pancreatitis.

Class of Variable	Variable	Odds Ratio	95% Confidence Interval
**Age**	Age (per 10 year increase)	**1.5**	1.3 – 1.8
**Gender**	Female	1.0	reference
	Male	0.8	0.4 – 1.3
**Race**	Non-White	1.0	reference
	White	**2.3**	1.2 – 4.6
**Ethnicity**	Non-Hispanic	1.0	reference
	Hispanic	1.0	0.4 – 2.4
**Geographic Region**	Northeast	1.0	reference
	Midwest	1.6	0.6 – 3.9
	South	0.8	0.4 – 1.6
	West	0.8	0.3 – 1.8
**Metropolitan Status**	Non-Metropolitan	1.0	reference
	Metropolitan	0.9	0.7 – 1.3
**Insurance Status**	Private	1.0	reference
	Public	0.9	0.6 – 1.3
	Other	0.8	0.4 – 1.6
**Urgency at Triage**	Not urgent	1.0	reference
	Urgent	1.5	0.8 – 3.1

## Discussion

This study provides the first U.S. estimates of ED visits for acute pancreatitis. From 1993 through 2003, there has been a steady increase in the absolute number and population rates of ED visits for acute pancreatitis. The number of estimated ED visits in 2003 (318,000) is approximately four times a recent U.S. government estimate of 80,000 annual cases [26]. Several European studies have described an increase in the incidence of acute pancreatitis in a variety of locations throughout Western Europe [1-3,27-30]. While these studies purport to describe incidence rates rather than ED visits – some use hospital admission data only, some include outpatient visits, and others include autopsy data – due to the lack of data from the United States and from EDs in general, they provide the most relevant context for interpreting our data. The overall rate of ED visits of 6.8/10,000/year that we describe here is higher than the incidence rates identified in most European studies, which report incidence rates from 1.5/10,000/year to 7.3/10,000/year in recent decades [1-3,27-34]. The 2003 peak of 10.9 ED visits per 10,000 U.S. population was higher than the peak annual rates in all the studies cited above.

There are several potential methodological explanations for these results. Our study is ED-based while some of the European studies cited above were based only on hospital admissions [2,3,27-30,32,34]. Extrapolating from our data, in which only 65% of ED visits ended in hospital admission, our calculated hospital admission rate would be significantly lower. Nevertheless, other European studies were prospective and included all healthcare facilities in the study area including outpatient clinics and the incidence rates reported by these investigators were notably lower than ours [33,35].

Our study did not impose strict criteria for the diagnosis of acute pancreatitis. The possibility that some of our sampled and selected ED visits did not have pancreatitis at all, or suffered exacerbations of chronic pancreatitis rather than episodes of acute pancreatitis is real. As noted above, though the NHAMCS reports a less than 2% error for items involving medical coding generally, the exact accuracy of pancreatitis diagnoses in the database is unknown. This problem of assuring accurate diagnosis was encountered in three large registry-based European studies with the capacity for chart review and validation (which our study lacks due to the anonymous nature of the NHAMCS). These showed from 82% to 90% rates of accurate diagnosis and coding for cases entered as acute pancreatitis [2,3,28]. All of these registries contained inpatient admissions, and so benefited from a sometimes lengthy hospital stay to clarify diagnosis – a luxury not afforded by the ED. In this respect, it is reassuring for our data that the National Hospital Discharge Summary, the largest national database for inpatient admissions in the U.S. has similarly documented a rise in hospital admissions for acute pancreatitis from 108,000 in 1987 to 224,000 in 2003 and that the 2003 figure for hospital admissions correlates closely with the number which would be computed from our ED visit and hospital admission rates [36,37]. Because of its anonymous nature, the NHAMCS database does not allow identification of repeat ED visits by the same patient for the same episode of acute pancreatitis. This may be mitigated somewhat by the inclusion of each hospital in the sampling frame for only one month out of approximately every 15, and is likely to have a small impact at most. While the inclusion of sample records without true acute pancreatitis or the capture of multiple visits for the same episode of acute pancreatitis would artificially elevate the observed caseload, one of the European studies cited documented capture of only 76% of a cohort of known pancreatitis patients by the national database, a source of error that would offset the over counting in terms of total numbers of cases [3]. Another study has shown that acute pancreatitis case selection based on reliance on diagnostic lists as in our study misses a large quantity of cases [38]. Despite these known limitations of registry-based studies and the difficulty of precisely validating NHAMCS for this particular cohort of cases, our selection criteria fell well within the standard methodology used to establish the literature in this field, and indeed were quite conservative.

Thus, we believe that methodologic reasons alone are unlikely to explain the high ED visit rates we describe, which are significantly higher than most of the incidence rates reported by European investigators. While NHAMCS itself does not allow calculation of incidence rates, we consider a higher incidence rate of acute pancreatitis in the U.S. to be the simplest explanation for the relatively high ED visit rate in this country. Methodological differences between our study and the numerous European studies cited cannot explain the observed upward trend in the overall number and rate of ED visits for acute pancreatitis in the U.S., nor can they explain differences in visit rates between subgroups within our study. Our selection criteria did not change for the different years of the study period, were applied uniformly, and no significant changes occurred in diagnostic modalities used in acute pancreatitis during the study period.

The rate of ED visits for acute pancreatitis among U.S. blacks was significantly higher than that among whites throughout the study period. To our knowledge, this is the first time that this racial disparity has been described in a large-scale nationwide study. This study is unable to further investigate the cause of this race disparity since the etiology of each case and whether or not it was a recurrent episode is not known. Because we cannot track the etiology of acute pancreatitis in any of our study subjects and do not know how many of the cases were recurrent, neither an explanation nor a means of rectifying this important racial disparity is possible. However, we believe that these results provide a compelling argument for future research on this topic.

Blacks were significantly less likely to be admitted than whites when controlling for age, insurance type, hospital location, and urgency at triage. This finding deserves particular attention given the high population rate of ED visits for acute pancreatitis for blacks, and has several possible explanations. Blacks in our study may have presented with milder pancreatitis than whites. Some studies have suggested that minority patients are more likely to use the ED as a source of primary care and more likely to present with non-urgent conditions [39]. While the NHAMCS does not provide many objective measures of disease severity, our observed disparity in admission likelihood persisted when we controlled for urgency at triage. Hospital differences may account for the disparity – hospitals treating blacks may be overcrowded, understaffed, or poorly funded. These hospitals may not discriminate between the black and white patients they treat, but because their patient population is disproportionately black, this phenomenon may produce the kinds of disparity we observed [40,41]. Patient decisions cannot be assessed with our data, and blacks in our study population may have declined admission in equivocal circumstances, while whites were more prone to accept. Finally, racial discrimination cannot be excluded and race-based differences in treatment and disposition of a variety of types of patients have been reported [42-44]. Variations in ethnicity, insurance type, urban setting, or U.S. region did not correlate with decision to admit.

The overall admission rate in our study was 65%. We are not aware of prior studies that have examined acute pancreatitis from the perspective of the ED so there are little (if any) data with which to compare this figure. Some European investigators have implied that in their study populations essentially all patients diagnosed with pancreatitis, even those diagnosed as outpatients, were referred for hospital admission [35]. We do not know how many of the people discharged from the ED in our study failed outpatient management and returned for admission. The very fact that the number of cases admitted directly from the ED to intensive care units (ICUs) is too small for reliable statistical extrapolation, illustrates the relative infrequency of initial admission to the ICU. This is in spite of estimates that between 14 and 20% of acute pancreatitis cases are considered to be "severe" [26,30,34]. We do not know how many of our admitted patients went on to be admitted to the ICU during their hospital stay, and at what point in the stay that might have happened, nor if initial admission to the ICU would have had any positive impact on the care of patients with this type of hospital course. All of these questions merit study and our data provide a foundation on which to build such studies. In the meantime, however, our data suggest that our population suffered from mild pancreatitis at the time of presentation, that previous estimates of the percentage of acute pancreatitis cases becoming severe are overstated, or that patients with severe pancreatitis are frequently admitted to low acuity inpatient units, as indeed has been noted by others [27]. In light of this latter possibility, it is reassuring that the one piece of information that we have available to us that is associated with both disease severity and prognosis – age – was independently associated with an increased likelihood of admission to the hospital (as noted, ICU admission numbers were too small for this type of analysis) [45,46].

Overall, diagnostic imaging was rarely used during ED visits for acute pancreatitis. CT scanning can be helpful in establishing the diagnosis of pancreatitis when it is in doubt and may be prognostically useful, though it is more frequently reserved for analyzing complications of severe acute pancreatitis such as necrosis, abscess, or pseudocyst [47,48]. Ultrasound is less sensitive for confirming the diagnosis of acute pancreatitis, but by detecting gallstones may suggest a biliary etiology, though this distinction is seldom important to make in the ED [49]. Infrequent use of these tests in the ED is probably appropriate.

Our data yield a variety of interesting questions that are beyond the scope of this study. For example, NHAMCS data do not allow us to evaluate the etiology of each attack of acute pancreatitis. The development of the *International Classification of Disease, 10^th ^Revision, Clinical Modification*, which features distinct codes for alcoholic and biliary pancreatitis will likely aid future investigators in this regard. We could not determine whether a case represented a first attack or a recurrent episode, a limitation of the anonymous nature of the NHAMCS and one that could be addressed by a prospective audit. Likewise, we could not evaluate mortality or any other outcomes beyond the ED, nor could we analyze care after admission to the hospital. Nationwide practice patterns regarding length and cost of hospital stay, rates of ICU admission, the use of antibiotics, the use of invasive therapies such as endoscopic retrograde cholangiopancreatography, percutaneous drainage, or surgery, and the use of enteral versus parenteral nutrition in inpatients with acute pancreatitis are essentially unknown. A large-scale study using nationwide inpatient data could address these large knowledge gaps in this important but relatively understudied condition.

## Conclusion

Our study provides the most complete epidemiologic picture to date of acute pancreatitis in the U.S. ED visits for acute pancreatitis are rising, and ED visit rates are higher among blacks than whites. Analgesics are not administered during many ED visits. Diagnostic imaging in the ED is rare. Most patients are admitted to the hospital, with greater likelihood of admission among whites than blacks. This study is the first to describe a recent increase in pancreatitis cases in U.S. EDs, the first to note a marked racial disparity in the rates of ED visits for acute pancreatitis, and the first to note a race-associated disparity in hospital admission patterns. We hope these data will stimulate further research into the causes of these important findings.

## Competing interests

The author(s) declare that they have no competing interests.

## Authors' contributions

PJF conceived of the study, designed the analysis, and drafted the manuscript; CFC provided expert advice on pancreatitis and aided in the preparation of the manuscript; NSH assisted in the study design and the preparation of the manuscript; AJP participated in the study design, performed the statistical analysis, and contributed particularly to the Methods section of the manuscript; CAC conceived of the study, and supervised the study design, statistical analysis, and preparation of the manuscript.

## Pre-publication history

The pre-publication history for this paper can be accessed here:


